# WHAT MATTERS IN HEALTH CARE TO OLDER NATIVE HAWAIIANS?

**DOI:** 10.1093/geroni/igae098.2395

**Published:** 2024-12-31

**Authors:** Miquela Ibrao, Rachel Burrage, Shelley Muneoka, Keilyn Kawakami, Tarin Tanji, Leslie Tanoue, Kathryn Braun

**Affiliations:** University of Hawaiʻi at Mānoa, Honolulu, Hawaii, United States; University of Hawaiʻi at Mānoa, Honolulu, Hawaii, United States; University of Hawaiʻi at Mānoa, Honolulu, Hawaii, United States; University of Hawaiʻi at Mānoa, Honolulu, Hawaii, United States; Rush University Medical Center, Chicago, Illinois, United States; ALU LIKE, Inc., Honolulu, Hawaii, United States; University of Hawaii, Honolulu, Hawaii, United States

## Abstract

The formal assessment of What Matters for older adults is often done in a medical setting through the acquisition of legal forms to prepare for end-of-life care. For many indigenous elders, however, these forms may appear impersonal or culturally insensitive. What Matters should go beyond end-of-life planning and include preferences for how older adults live their daily lives, which may include preferences for dwelling status and relationships, in addition to medical care. For Native Hawaiian elders, cultural differences exist that impact their aging values and priorities. A secondary qualitative analysis of 20 interviews was conducted in partnership with Hā Kūpuna National Resource Center for Native Hawaiian Elders using a transformative lens to explore themes on What Matters to Native Hawaiian kūpuna as they age. Deductive coding was informed by guidelines on What Matters according to the Institute of Healthcare Improvement’s 4Ms Framework. Inductive codes identified themes specific to Native Hawaiian kūpuna as part of their culture. NVIVO software was used. Themes identified on What Matters to Native Hawaiian older adults included: 1) incorporation of cultural traditions into healthcare routines; 2) a strong sense of family as part of the decision-making process; 3) a preference for home-based care at the end-of-life; and 4) the importance of provider relationship building. An infographic with education and recommendations for healthcare providers was developed using findings from the study. Our interview, coding, and dissemination processes may be of interest to researchers working with other minority groups.

